# Longitudinal Ultrasound Monitoring of Peripheral Muscle Loss in Neurocritical Patients

**DOI:** 10.3390/jimaging11090297

**Published:** 2025-09-01

**Authors:** Talita Santos de Arruda, Rayssa Bruna Holanda Lima, Karla Luciana Magnani Seki, Vanderlei Porto Pinto, Rodrigo Koch, Ana Carolina dos Santos Demarchi, Gustavo Christofoletti

**Affiliations:** 1 Institute of Health, School of Medicine, Federal University of Mato Grosso do Sul, UFMS, Campo Grande 79060-900, Brazil; talita.arruda@ufms.br (T.S.d.A.); rayssa.lima_@hotmail.com (R.B.H.L.); karla.magnani@ufms.br (K.L.M.S.); portovanderley@gmail.com (V.P.P.); 2 Hospital Maria Aparecida Pedrossian, Campo Grande 79080-190, Brazil; rodrigo@pullmonar.com.br; 3 Course of Physiotherapy, Anhanguera-Uniderp University, Campo Grande 79003-010, Brazil; anacarolinademarchi@gmail.com

**Keywords:** ultrasonography, muscle weakness, critical care outcomes, hospitalization, muscular atrophy

## Abstract

Ultrasound has become an important tool that offers clinical and practical benefits in the intensive care unit (ICU). Its real-time imaging provides immediate information to support prognostic evaluation and clinical decision-making. This study used ultrasound assessment to investigate the impact of hospitalization on muscle properties in neurocritical patients and analyze the relationship between peripheral muscle changes and motor sequelae. A total of 43 neurocritical patients admitted to the ICU were included. The inclusion criteria were patients with acute brain injuries with or without motor sequelae. Muscle ultrasonography assessments were performed during ICU admission and hospital discharge. Measurements included muscle thickness, cross-sectional area, and echogenicity of the biceps brachii, quadriceps femoris, and rectus femoris. Statistical analyses were used to compare muscle properties between time points (hospital admission vs. discharge) and between groups (patients with vs. without motor sequelae). Significance was set at 5%. Hospitalization had a significant effect on muscle thickness, cross-sectional area, and echogenicity in patients with and without motor sequelae (*p* < 0.05, effect sizes between 0.104 and 0.475). Patients with motor sequelae exhibited greater alterations in muscle echogenicity than those without (*p* < 0.05, effect sizes between 0.182 and 0.211). Changes in muscle thickness and cross-sectional area were similar between the groups (*p* > 0.05). Neurocritical patients experience significant muscle deterioration during hospitalization. Future studies should explore why echogenicity is more markedly affected than muscle thickness and cross-sectional area in patients with motor sequelae compared to those without.

## 1. Introduction

Acute brain injuries are defined as sudden damage to the brain caused by internal or external events. These conditions typically develop rapidly and can lead to severe neurological impairment. Neurological events commonly affect both cognitive and motor functions, with patients experiencing long-term complications and social reintegration challenges. Recovery is typically prolonged and requires multidisciplinary rehabilitation to enhance functional outcomes and quality of life [1].

Although advances in intensive care units (ICUs) have improved survival rates, many patients face significant impairments. Common complications include cognitive dysfunction, psychological distress, and ICU-acquired muscle weakness [2,3]. ICU-acquired muscle weakness stems from a combination of factors, such as systemic inflammation, prolonged immobilization, malnutrition, and certain medications. This can limit mobility, hinder ventilator weaning, and compromise long-term recovery [4,5].

Diagnostic strategies for ICU-acquired muscle weakness include invasive methods, such as muscle biopsy and electroneuromyography, as well as non-invasive clinical assessments of muscle strength. However, invasive methods are costly, technically demanding, and not routinely available in most health care settings. Clinical evaluations require patient cooperation and active participation, which are frequently compromised in patients with acute neurological conditions. These limitations highlight the need for accessible, objective, and patient-independent diagnostic alternatives.

Peripheral muscle ultrasound has emerged as a promising point-of-care tool that offers several advantages in ICU settings [6]. It is a noninvasive, repeatable, and cost-effective technique that can be performed at the bedside, offering real-time insights into muscle morphology. Muscle ultrasound typically assesses three primary variables: muscle thickness, cross-sectional area, and echogenicity, which collectively reflect the muscle mass and structural integrity [6].

Muscle thickness refers to the distance between the superficial and deep fasciae of the muscle and is measured in a linear fashion. It is considered a direct indicator of muscle mass and is used to track ICU-acquired muscle weakness. A decrease in muscle thickness over time reflects catabolic processes and disuse atrophy, which are common in neurocritical patients [7,8,9,10]. The cross-sectional area represents the total muscle surface when the ultrasound transducer is placed perpendicular to the muscle belly. The cross-sectional area is a more volumetric measurement that provides a robust estimation of the overall muscle size and correlates strongly with muscle strength and functional capacity. A reduction in the cross-sectional area has been associated with both acute muscle loss and poor rehabilitation outcomes. Echogenicity refers to the grayscale intensity of muscle tissue on ultrasound images. Healthy muscles typically appear as dark/hypoechoic structures with characteristic horizontal fibrous lines. Increased echogenicity suggests pathological changes such as fatty infiltration, edema, fibrosis, or inflammatory processes, which impair muscle quality and function. Elevated echogenicity has been linked to decreased muscle contractility and poor clinical outcomes [10].

**Figure 1:**
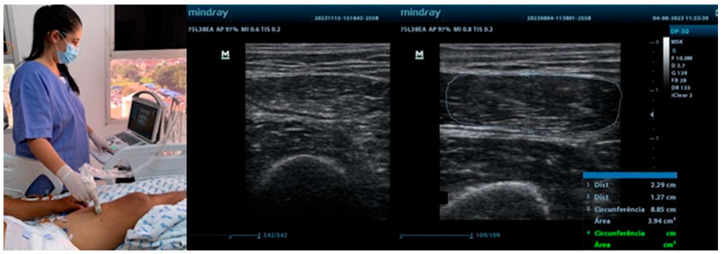
Ultrasound assessment in the ICU.

The quadriceps femoris, rectus femoris, and biceps brachii muscles are among the most frequently assessed sites in ICU patients. Their selection is primarily due to their easy accessibility for ultrasonography, their key role in mobility and overall functional performance, and their high sensitivity to the deleterious effects of immobilization and critical illness. The quadriceps femoris and rectus femoris muscles are essential for lower-limb strength, gait, and the ability to perform activities of daily living. Differently, the biceps brachii muscle provides insight into upper-limb function and overall muscular health. Evaluating these muscles enables the healthcare team to monitor both upper- and lower-limb status, offering a comprehensive assessment of peripheral muscle changes throughout hospitalization [9,10]. Figure 1 illustrates the use of ultrasound in neurocritical patients in the ICU, emphasizing aspects of muscle thickness, cross-sectional area, and echogenicity of peripheral muscles.

Recent studies have demonstrated that muscle mass loss detected by ultrasound is associated with decreased muscle strength, functional impairment, and reduction in muscle size [8,9,10,11,12]. Muscle ultrasound also exhibits excellent inter- and intra-rater reliability, reinforcing its potential as a robust monitoring tool in critical care [11,12,13,14,15]. As a result, there is a growing interest in using muscle ultrasound to track the progression of muscle atrophy and to potentially guide early interventions in patients with ICU-acquired muscle weakness.

Despite these promising findings, several gaps remain in the literature. It is unclear which ultrasound parameters are most predictive of clinically meaningful outcomes, such as muscle strength and functional capacity. Furthermore, there is a paucity of longitudinal studies that have specifically focused on neurocritical patients who present with a unique combination of neurological and systemic challenges. The correlation between muscle morphology and functional recovery over different follow-up periods remains insufficiently explored and is somewhat inconsistent in the existing literature. Factors such as the influence of a specific neurological diagnosis, the timing and frequency of muscle assessments, and the potential confounding effects of systemic complications have not been adequately addressed.

Given this gap, the present study aimed to use ultrasound to comprehensively evaluate the impact of hospitalization on peripheral muscle properties in neurocritical patients and investigate the association between ultrasound findings, motor sequelae, and functional outcomes throughout a hospital stay. We hypothesized that hospitalization in neurocritical patients leads to significant alterations in the peripheral muscle properties detectable by ultrasound and that these changes are associated with worse motor outcomes and reduced functional status at discharge.

## 2. Methods

This longitudinal study included two parallel groups: one group of individuals with motor sequelae caused by acute brain injuries and another group without motor sequelae. Between 2023 and 2024, a total of 683 patients were assessed for eligibility. The study was conducted at Santa Casa Hospital, located in Campo Grande, Brazil. Ethical approval was obtained from the Research Ethics Committee of the Federal University of Mato Grosso do Sul (protocol number 5381.783). Informed consent was obtained from all patients prior to participation.

**Figure 2:**
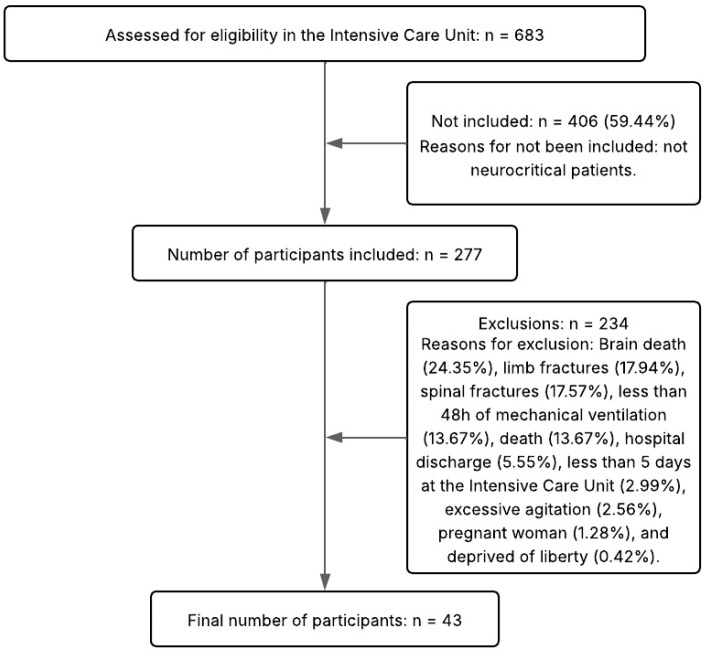
Flow diagram of the study.

The inclusion criteria were individuals with acute brain injuries within 48 h of hospitalization, aged 18 years or older, and of both sexes. The exclusion criteria were severe agitation (patients with physical and verbal aggression), an ICU stay of less than five days, patients with amputation, fractures, or deformities of the limbs, burns, patients who received palliative care, those who had died during hospitalization, and individuals undergoing investigation of brain death. A total of 683 patients were screened, of which 406 did not meet the eligibility criteria. Because of the inclusion and exclusion criteria, 234 were subsequently excluded. Figure 2 details the flow of participant selection and monitoring.

### 2.1. Evaluative Assessment

Within the first 48 h of the patient’s stay at the ICU, informed consent was obtained. At this time, the patient data sheet was completed, and the Acute Physiology and Chronic Health Evaluation (APACHE II) score was recorded. Additional general data were collected, including personal and clinical information, the presence of prior morbidities and/or comorbidities, the use of previous medications, and the cause of the acute brain injury. The Glasgow Coma Scale [16] was used to assess the level of consciousness, whereas the ICU Mobility Scale [17] was used to evaluate functional capacity. The ICU Mobility Scale ranges from 0 to 10, with higher scores indicating greater functional ability.

Muscle evaluations were performed at two time points: within 48 h of hospital admission and at discharge. Ultrasonography was used to assess the biceps brachii and the rectus femoris component of the quadriceps femoris muscle, focusing on morphological parameters. All assessments were conducted by a researcher experienced in peripheral muscle ultrasound and trained in image acquisition. The ultrasound device used in this study was a Mindray^®^ N-30 (Mindray Bio-Medical Electronics Co., Ltd., Shenzhen, China).

For the ultrasound evaluation, the patients were placed in the supine position with the head elevated to 30°. Bilateral imaging was performed for all muscles. A high-frequency linear-array transducer was used to obtain images of the muscles. Ultrasound settings were optimized and kept consistent across all assessments to ensure image quality and comparability. Muscle thickness, cross-sectional area, and echogenicity were evaluated, with care taken to avoid excessive pressure on the skin to prevent compression artifacts. The assessments followed the standardized image acquisition protocol described by Arts et al. [18].

For the biceps brachii, the transducer was positioned at two-thirds of the craniocaudal distance between the acromion and the cubital fossa on both sides. For the rectus femoris, the muscle thickness, cross-sectional area, and echogenicity were assessed at the lower middle third of the distance between the anterosuperior iliac spine and upper edge of the patella. The cross-sectional area and echogenicity were measured using manual tracing techniques, and the echogenicity was calculated based on the average of three images obtained at each time point.

### 2.2. Statistical Analysis

The results of this study are presented using both descriptive and inferential statistics. Continuous variables are expressed as means and standard deviations. Categorical variables are presented as absolute and relative frequencies, allowing for the assessment of proportional distributions within the sample.

To compare peripheral muscle ultrasound measurements across the different assessment time points, appropriate statistical tests were selected based on the data distribution. When the data followed a normal (parametric) distribution, comparisons were performed using a repeated-measures ANOVA. In cases in which the data did not meet the assumptions of normality, Friedman’s test was applied. Correlations between muscle changes and clinical outcomes (including motor sequelae and functional status) were analyzed using Spearman’s rank correlation coefficient. The strength and clinical relevance of the correlations were interpreted based on the classification proposed by Akoglu [19].

The Supplementary Materials (Tables S1–S3) contain the raw data on participants’ clinical characteristics, along with their baseline and follow-up ultrasound measurements. All statistical analyses were conducted considering a two-tailed significance level of 5%. The effect sizes were calculated and reported for all statistically significant comparisons.

## 3. Results

The sample consisted of patients with severe neurocritical conditions associated with acute brain injuries. Of the total participants, 70% required neurosurgical intervention, which included procedures of decompressive craniectomy, evacuation of hematomas, and placement of intracranial pressure monitoring devices. The remaining 30% were managed conservatively with clinical measures, without the need for surgical procedures.

The estimated mortality risk differed between the treatment groups: patients managed conservatively presented a mortality risk of 38%, while those who underwent neurosurgical procedures had a post-surgical ICU mortality risk of 30%. Regarding hospitalization, the mean length of stay in the ICU was 10.0 ± 4.0 days, indicating prolonged critical care support in both groups. The mean total hospital length of stay was 21.0 ± 18.4 days, demonstrating substantial variability, which may be associated with individual recovery trajectories, complications, or the need for extended rehabilitation. The epidemiological and clinical characteristics of the study population are summarized in Table 1.

Table 2 shows the division of the participants into groups with and without motor sequelae. Comparisons between groups revealed significant differences in muscle thickness (left rectus femoris) and echointensity (right and left rectus femoris). A significant time effect was observed for all variables, except for the thickness of the left rectus femoris. The group–time interaction was not significant for any variable. However, the mean changes over time showed a pattern of greater decline in the group with motor sequelae than in the group without sequelae.

At ICU admission, the group with motor sequelae had a functional capacity score of 2.3 ± 1.7 points (95% CI: 1.6–3.0), whereas the group without sequelae scored 4.8 ± 2.7 points (95% CI: 3.5–6.2). By hospital discharge, functional capacity improved in both groups, with the sequelae group reaching a mean score of 4.8 ± 1.9 points (95% CI: 4.0–5.6) and the non-sequelae group achieving a significantly higher score of 8.6 ± 1.4 points (95% CI: 7.8–9.3). A repeated measures analysis indicated a significant main effect of time (*p* = 0.001; effect size: 0.636) and group (*p* = 0.001; effect size: 0.489) but no significant group–time interaction (*p* = 0.086).

**Figure 3:**
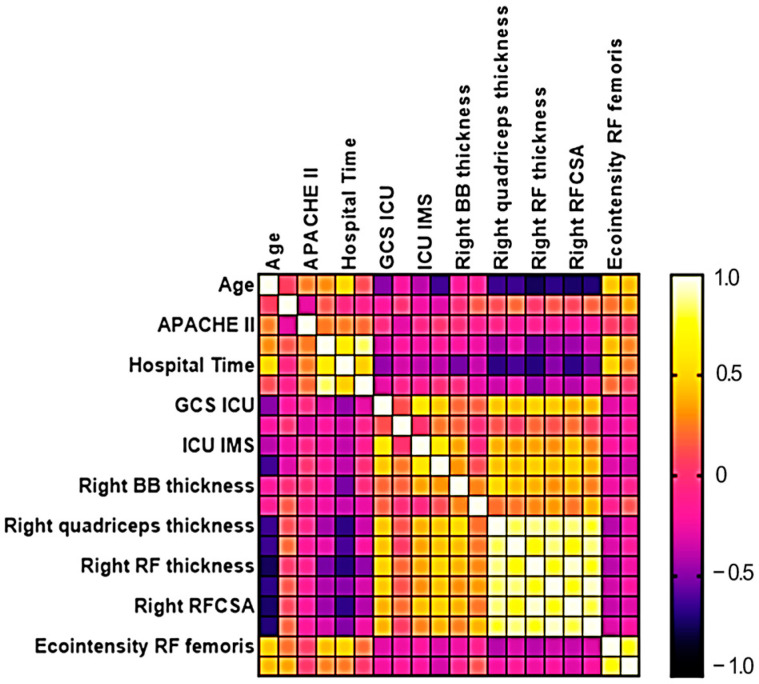
Matrix correlation analysis between ultrasound variables and clinical aspects of neurocritical patients.

The results of the correlation matrix analysis are shown in Figure 3. A strong negative correlation was observed between age and right rectus femoris muscle thickness (r_s_ = −0.751) and right rectus femoris cross-sectional area (r_s_ = −0.727). A moderate negative correlation was found between age and several muscle ultrasound parameters, including right quadriceps muscle thickness (r_s_ = −0.643), left quadriceps muscle thickness (r_s_ = −0.647), left rectus femoris muscle thickness (r_s_ = −0.674), and left rectus femoris cross-sectional area (r_s_ = −0.700). A strong positive correlation was identified between ICU length of stay and the duration of mechanical ventilation (r_s_ = 0.825). A moderate positive correlation was observed between total hospital length of stay and ICU length of stay (r_s_ = 0.599). Regarding functional capacity, a moderate negative correlation was found between age and functional capacity scores (r_s_ = −0.635), suggesting that older patients tended to have lower functional capacity at discharge.

Hospital length of stay showed moderate negative correlations with the following muscle measurements: right biceps brachii thickness (r_s_ = −0.535), right quadriceps thickness (r_s_ = −0.675), left quadriceps thickness (r_s_ = −0.617), right rectus femoris thickness (r_s_ = −0.692), right rectus femoris cross-sectional area (r_s_ = −0.673), and left rectus femoris cross-sectional area (r_s_ = −0.510). These findings suggest that prolonged hospitalization was associated with greater muscle mass loss, particularly in the lower limbs.

## 4. Discussion

This study identified significant differences in the clinical characteristics and muscle ultrasound measurements between patients with and without motor sequelae. These differences likely reflect the impact of critical conditions and prolonged immobilization on patient outcomes. Notably, patients with motor sequelae were older and had a higher body mass index than those without sequelae, which may have contributed to worse functional recovery and a higher prevalence of comorbidities.

The assessment of the biceps brachii muscle using ultrasound has scarcely been explored in the literature [20,21]. In one of the few available studies, the authors used ultrasound to monitor muscle mass and compared it with bioimpedance measurements. They found a progressive decrease in muscle mass by the tenth day of hospitalization, supporting ultrasound as a useful tool for tracking muscle atrophy in neurocritical patients [20]. Similarly, another study found that ultrasound measurements of the biceps brachii and anterior thigh were better predictors of lean tissue loss in edematous patients than other methods [21]. In our study, a significant atrophy of the biceps brachii muscle thickness was observed in both groups with and without motor sequelae, when comparing hospital admission and hospital discharge.

Recent prospective studies involving critically ill patients have reported a gradual reduction in the cross-sectional area of the biceps brachii [21,22,23]. These changes were associated with handgrip strength, functional status, and mortality on days five and seven of hospitalization. Differently, other studies have not found a clear association between biceps brachii ultrasound findings and ICU-acquired weakness. The utility of assessing biceps brachii muscle thickness by ultrasound remains under debate, indicating a need for further research [24,25,26,27].

A limited number of studies have focused exclusively on neurocritical patients using peripheral muscle ultrasound to assess both admission and discharge outcomes [26,27]. In our study, the mean loss of quadriceps muscle thickness was 23% in the sequelae group and 18% in the non-sequelae group. Comparable findings were reported in a study of COVID-19 patients admitted to the ICU, which showed a reduction of 18.6% in quadriceps muscle thickness and a 30.1% decrease in the rectus femoris cross-sectional area by the tenth day of hospitalization [28]. Another study in ICU patients reported muscle thickness losses of 12% and 15% in the right and left quadriceps, respectively [26]. Previous studies have linked greater quadriceps muscle loss, as measured by ultrasound, to poorer clinical outcomes and reduced muscle strength in critically ill patients requiring mechanical ventilation [9,28,29].

Puthucheary et al. [29] reported a significant reduction in the rectus femoris cross-sectional area by the seventh day of hospitalization. In comparison, our study found an average cross-sectional area loss of 19% in the sequelae group and 21% in the non-sequelae group. Differences in echogenicity were observed between the sequelae and non-sequelae groups at different time points. Similarly, Hayes et al. [30] observed a reduction in quadriceps muscle thickness in critically ill patients but reported no changes in rectus femoris echogenicity by the tenth day of hospitalization. This may be explained by the patient profile in their study, which included younger individuals, patients with acute but non-chronic conditions, and those with relatively short ICU stays. Several studies have demonstrated that increased muscle echogenicity is associated with decreased muscle function and strength, regardless of muscle mass loss [31,32,33]

Consistent with our findings, Puthucheary et al. [29] identified a significant association between changes in rectus femoris cross-sectional area and length of ICU stay. Similarly, Parry et al. [7] found a moderate correlation between the rectus femoris cross-sectional area and functional capacity on the tenth day of ICU stay. Other authors have reported strong associations between ICU length of stay, handgrip strength, and functional scores, but not with ultrasound findings [24,25,26,27]. Although these results are interesting, it is important to recognize that correlations do not imply causation. Nevertheless, they are valuable because they can identify meaningful relationships and generate hypotheses for future research, guiding further investigation into the underlying mechanisms and potential interventions.

In our study, echogenicity differed between patients with and without motor sequelae, whereas muscle thickness and cross-sectional area were affected similarly in both groups. We hypothesize that the difference in echogenicities may be related to muscle hypotonia, a common phenomenon in patients with acute motor sequelae, which could influence muscle echogenicity. Muscle thickness and cross-sectional area may have been similarly affected because the duration of hospitalization was relatively short (average hospitalization time of 29.0 days for the sequelae group and 22.0 days for the non-sequelae group). A longer hospital stay would likely have a greater impact on muscle thickness and cross-sectional area. Further studies are needed to confirm this hypothesis [34,35].

As a limitation, we acknowledge that inter- and intra-rater reliability were not formally assessed in this study. However, all ultrasound evaluations were performed by an experienced examiner, following a standardized protocol previously validated in the literature. This approach aimed to minimize variability and ensure consistency across measurements. While this represents a limitation, we believe that it does not significantly compromise the internal validity of the findings, given the controlled and uniform assessment procedures.

## 5. Conclusions

Neurocritical patients experience significant muscle changes during hospitalization. While all patients showed muscle alterations, those with motor sequelae demonstrated greater changes in muscle echogenicity than patients without sequelae. In contrast, the changes in muscle thickness and cross-sectional area were similar between the groups. Future research should investigate why echogenicity appears to be more markedly affected than muscle thickness and cross-sectional area in patients with motor sequelae.

Based on the ultrasound findings, we recommend implementing early rehabilitation strategies in ICU patients to mitigate muscle atrophy and preserve functional capacity. Early mobilization and targeted interventions should be guided by ultrasound assessments to identify patients at a higher risk of muscle deterioration and optimize recovery outcomes.

## Figures and Tables

**Table 1:** Clinical characteristics of the participants.

Variables		All Participants	Participants with Motor Sequelae	Participants Without Motor Sequelae	*p*
Sample size, *n*		43	25	18	0.286
Age (yrs)		45.0 ± 17.0	51.0 ± 14.0	37.0 ± 17.0	0.008
Glasgow (score)		13.8 ± 1.9	14.0 ± 2.0	14.0 ± 1.0	0.209
Treatment (%)	Surgical	70.0	76.0	60.0	0.331
	Conservative	30.0	24.0	40.0	
Presence of comorbidity (%)	Yes	58.0	68.0	44.0	0.209
	No	42.0	32.0	56.0	
Weight (kg)		75.0 ± 16.0	79.5 ± 16.0	70.5 ± 12.0	0.051
BMI (kg/m^2^)		27.0 ± 5.0	29.0 ± 5.0	23.0 ± 3.0	0.006
APACHE II (score)		21.0 ± 5.5	20.5 ± 5.5	22.0 ± 6.0	0.438
Length of stay in the ICU (days)		10.0 ± 4.0	10.0 ± 3.0	9.0 ± 4.0	0.270
Hospital stay time (days)		21.0 ± 18.4	29.0 ± 19.0	22.0 ± 16.0	0.272

**Table 2:** Comparative analysis of the peripheral muscle ultrasound of the groups.

Variables	Groups	Assessments		Main Effect (*p* and Effect Size)		
		Admission	Discharge	Group	Time	Interaction
Right biceps brachii thickness	With sequelae	2.6 ± 0.7	2.3 ± 0.6	*p* = 0.135	*p* = 0.030 ES: 0.110	*p* = 0.185
	Without sequelae	2.3 ± 0.5	2.2 ± 0.4			
Left biceps brachii thickness	With sequelae	2.6 ± 0.7	1.9 ± 0.5	*p* = 0.928	*p* = 0.001 ES: 0.303	*p* = 0.053
	Without sequelae	2.4 ± 0.5	2.1 ± 0.6			
Right quadriceps thickness	With sequelae	2.4 ± 0.8	1.8 ± 0.6	*p* = 0.070	*p* = 0.001 ES: 0.492	*p* = 0.716
	Without sequelae	2.7 ± 0.5	2.2 ± 0.5			
Left quadriceps brachii thickness	With sequelae	2.4 ± 0.8	1.9 ± 0.7	*p* = 0.203	*p* = 0.001 ES: 0.475	*p* = 0.981
	Without sequelae	2.6 ± 0.5	2.1 ± 0.5			
Right rectus femoris thickness	With sequelae	1.3 ± 0.4	1.1 ± 0.3	*p* = 0.285	*p* = 0.001 ES: 0.221	*p* = 0.514
	Without sequelae	1.4 ± 0.2	1.2 ± 0.3			
Left rectus femoris thickness	With sequelae	1.2 ± 0.3	1.1 ± 0.3	*p* = 0.040 ES: 0.099	*p* = 0.062	*p* = 0.875
	Without sequelae	1.4 ± 0.2	1.4 ± 0.3			
Right rectus femoris cross-sectional area	With sequelae	4.0 ± 1.6	3.0 ± 1.2	*p* = 0.445	*p* = 0.001 ES: 0.292	*p* = 0.184
	Without sequelae	4.1 ± 1.1	3.5 ± 0.9			
Left rectus femoris cross-sectional area	With sequelae	3.6 ± 1.5	3.2 ± 1.3	*p* = 0.159	*p* = 0.004 ES: 0.185	*p* = 0.891
	Without sequelae	4.2 ± 1.0	3.7 ± 1.1			
Right rectus femoris echogenicity	With sequelae	69.9 ± 23.9	64.1 ± 23.2	*p* = 0.002 ES: 0.211	*p* = 0.035 ES: 0.104	*p* = 0.935
	Without sequelae	51.0 ± 12.7	45.7 ± 14.0			
Left rectus femoris echogenicity	With sequalae	72.8 ± 22.6	64.3 ± 20.8	*p* = 0.004 ES: 0.182	*p* = 0.002 ES: 0.207	*p* = 0.878
	Without sequelae	57.3 ± 14.9	48.0 ± 14.3			

Note. ES: effect size.

## Data Availability

The original contributions presented in the study are included in the article and Supplementary Materials. Further inquiries can be directed to the corresponding author.

## Supplementary Materials

The following supporting information can be downloaded at https://www.mdpi.com/article/10.3390/jimaging11090297/s1: Table S1. Clinical Characteristics of Participants; Table S2. Baseline Ultrasound Data; Table S3. Follow-up Ultrasound Data.
