# Supplementary material for: Longitudinal Ultrasound Monitoring of Peripheral Muscle Loss in Neurocritical Patients
Source: J Imaging. 2025 Sep 1;11(9):297. doi: 10.3390/jimaging11090297 (PMC12470513; doi:10.3390/jimaging11090297)
Supplement: Supplementary file 1 [file jimaging-11-00297-s001.zip › jimaging-3773902-supplementary.pdf]

SUPPLEMENTARY MATERIAL – RAW DATA FROM THE STUDY

Table S1. Clinical Characteristics of Participants (Part 1)

Participant	Sex	Treatment	Comorbididty	Weight	BMI	APACHE II
3	M	CI	Tabagismo/ etilismo	55	18.8	28
7	F	CI	HAS/obesidade	81	31.95	16
8	M	CI	HAS/etilista	87	29	22
10	F	CI	x	79.8	28.6	14
15	M	CI	HAS	80	30.1	25
17	M	CI	obesidade	122	36.3	23
22	M	CO	x	82.5	26.3	15
24	F	CI	x	90	35.6	23
25	M	CI	x	77.6	27	25
26	F	CI	HAS	80	31.2	22
27	F	CI	HAS, obesidade	100	38.1	20
28	F	CO	HAS, asma	78.5	29.9	12
31	M	CI	HAS	83.1	25.9	26
33	M	CI	x	69	24.2	26
34	M	CI	HAS	66	24.2	28
35	M	CO	x	72	26.2	23
36	M	CO	x	64	21.4	21
46	M	CI	HAS, DM	76	27.2	32
48	M	CI	x	101	30.49	17
50	F	CI	x	84	29.76	21

52	F	CI	HAS	55.9	23.57	21
59	F	CI	x	72	28.12	20
60	M	CI	x	62	29	17
63	F	CI	HAS, tabagista	52.4	20.47	23
64	F	CI	transtorno depressivo	63.7	23.9	10
73	M	CO	x	90.1	27.5	24
74	M	CI	tabagista	89.2	30	19
75	M	CO	x	66	22.84	28
76	F	CO	HAS, DM	45	18.4	28
88	M	CI	etilismo	70.9	24.5	25
92	F	CO	hipertiroidismo	85.2	29.1	25
98	M	CI	x	78	23.4	31
93	M	CO	x	85	23.5	13
98	F	CI	HAS	94.5	36.9	16
99	M	CO	tabagismo + maconha	65	28	18
106	M	CO	Tabagismo	80.5	21.61	15
109	M	CI	Etilismo/DRC dialitico/ Drogadição	56	20.57	32
110	M	CI	Etilismo/Capróstata/Cardiopatia	74	24.16	23
116	F	CI	Depressão	54	22.52	21
125	F	CI	Bariatrica	92	38	12
128	F	CI	x	47.7	20.1	21
132	M	CO	x	68.5	22.8	11
134	M	CO	x	70	24	17

Table S1. Clinical Characteristics of Participants (Part 2)

Days VMI	Days Sed(h)	StatusventICU	GCSaltaICU	StatusventHOSP	IMSaltaICU	IMSaltaHOSP	MRCaltaICU	MRCaltaHOSP	HandICU	HandHOSP	Motor sequelae
12	48	TQT	11	VE	4	10	<48	>48	x	x	x
4	24	VE	11	VE	0	5	X	<48	x	11E	Hemiplegia D
11	48	TQT	10	VE	0	5	X	<48	x	x	plegia braquial D
13	48	TQT	5	VE	1	3	X	<48	x	x	hemiparesia E
10	120	TQT	11	VE	0	3	X	<48	x	10E	hemiparesia D
8	24	TQT	11	VE	3	7	X	>48	x	x	quadriparesia
9	48	TQT	14	VE	8	10	>48	48	26D;38E	31D; 34E	x
6	96	TQT	10	VE	3	5	X	<48	x	x	Hemiparesia E
7	48	VE	13	VE	3	3	X	36	24D; 0E	25D	plegia braquial E
5	120	TQT	10	VE	2	8	X	x	x	8E	hemiparesia D
6	48	TQT	10	VE	1	4	X	<48	x	8E	Hemiplegia D
7	24	TQT	11	VE	1	3	X	<48	x	12D	Hemiplegia E
8	24	TQT	8	VE	2	8	X	59	x	24D; 20E	x
9	120	TQT	8	VE	1	7	X	55	x	14D; 22E	x
4	48	VE	14	VE	3	7	X	47	22E	8E	x
10	192	TQT	10	VE	3	5	X	<48	x	x	x
6	24	TQT	10	VE	5	10	58	58	15D; 22E	15D; 22E	x
com trilogy	48	TQT	3	VE	1	3	X	x	x	x	hemiplegia
4	48	VE	14	VE	8	8	60	60	54D; 42E	60D; 54E	x
8	72	TQT	10	VE	3	4	X	<48	x	18D	hemiplegia E
com trilogy	72	TQT	10	TQT	3	8	<48	48	x	x	Hemiplegia D
10	72	TQT	11	TQT	5	10	36	60	5D; 5E	18D; 24E	x
3	48	TQT	3	VE	1	5	X	<48	x	x	Hemiplegia E
com trilogy	144	TQT	6	VE	1	5	X	<48	x	x	hemiparesia D
5	48	VE	14	VE	3	5	X	<48	x	7D	hemiplegia E
5	72	VE	15	VE	8	8	>48	>48	36D; 14 E	x	paresia em MSE

4	24	TQT	8	VE	3	3	X	x	x	x	quadriparesia
3	24	VE	10	VE	3	3	X	x	x	x	hemiparesia a direita
com trilogy	72	TQT	11	VE	3	3	X	x	x	x	hemiparesia a esquerda
8	96	VE	15	VE	8	8	56	60	50D;53E	52D;56E	x
5	120	TQT	11	TQT	4	8	<48	<48	x	x	quadriparesia
8	48	VE	14	VE	3	10	<48	<48	X	26D;32E	x
3	48	VE	14	VE	3	3	<48	<48	24D;16E	24D;16E	paresia em MID, AGITAÇÃO
8	24	TQT	11	VE	5	8	<48	43	X	10D;0E	hemiparesia a esquerda
5	48	VE	15	VE	1	10	58	60	42D;40E	66D;70E	x
5	24	VE	15	VE	5	10	<48	64	x	40D;38E	X
4	96	VE	12	VE	3	8	36	50	x	14D;18E	x
3	48	TQT	9	VE	1	5	<48	42	x	x	hemiparesia
10	8	TQT	11	VE	8	8	48	52	x	22D;5E	X
6	96	TQT	9	VE	3	3	<48	46	x	x	hemiparesia a direita
4	96	VE	15	VE	8	8	48	55	14D;14E	20D;20E	X
com trilogy	48	TQT	8	VE	3	8	48	56	x	30D;36E	X
3	72	VE	14	VE	10	10	58	60	38D;38E	38D;38E	X

Table S2. Baseline Ultrasound Data

RBiceps R-T	LBiceps R-T	RQuad-T	LQuad-T	RRF-T	LRF-T	RRF-CA	LRF-CA	RRF-Echoin	LRF-Echoin
2.61	3.52	2.1	2.07	1.11	1.02	2,57 (8,09)	3,12 (8,12)	n=21275; m=59.3	n=40836; m=79,7
3.54	3.73	3.49	4.05	1.73	1.81	5,05 (8,93)	5,30 (9,07)	n=24298; m=104,7	n=11034; m=88,8
4.37	4.14	4.1	3.15	1.93	1.63	7,32 (11,10)	4,37 (8.83)	n=21688; m=77.4	n=19325; m=53,5
3.12	3.22	2.82	3	2.08	1.48	6,35 (9,68)	4,84 (9,40)	n=20099; m=98,5	n=20922; m=91,9
2.47	2.8	2.66	2.14	1.43	1.31	4,49 (8,87)	4,05 (8,97)	n=39005; m=54,9	n=54584; m=59,9
2.93	3.41	3.8	4	2.38	2.29	7,36 (10,40)	7,60 (10,67)	n=22006; m=68,3	n=26179; m=76,1
2.74	2.68	3.52	3.61	1.93	1.93	6,63 (10,12)	6,83 (10,18)	n=37956; m=45,3	n=41179; m=51,6
3.88	3.67	2.96	3.47	1.67	1.82	5,34 (9,68)	6,14 (10,55)	n=23024; m=95,1	n=25538; m=79,9
2.73	2.91	2.79	2.34	1.08	1.06	3,52 (8,44)	3,16 (8,44)	n=20154; m=64,7	n=18275; m=60
1.78	2	2.24	2.17	1.21	1.11	3,59 (7,97)	2,59 (6,71)	n=14222; m=115.6	n=100.40; m=126.9
2.49	2.77	1.82	1.23	1.52	0.94	4,56 (8,71)	2,14 (7,26)	n=15917; m=111,5	n=8891; m=125,8
2.82	2.23	1.41	1.6	0.66	0.68	1,75 (7,20)	2,66 (7,84)	n=11303; m=76,4	n=14313; m=84,6
1.28	1.45	1.81	2.04	1.01	1.2	2,63 (8,09)	3,61 (8,60)	n=37383; m=86	n=48985; m=77
2.27	2.1	3.05	3.2	1.49	1.61	4,40 (8,58)	5,25 (9,25)	n=20799; m=56,6	n=24338; m=58,8
1.91	2.5	2.23	2.35	1.27	1.2	3,98 (8,76)	3,17 (8,59)	n=16895; m=47,1	n=13774; m=71,7
2.56	2.07	2.37	1.97	1.03	1.5	3,36 (9,48)	3,93 (8,73)	n=43777; m=44,7	n=54385; m=51,9
2.13	2	2.6	2.42	1.24	1.33	3,38 (8,23)	4,03 (8,61)	n=20294; m=42,9	n=24110; m=32,7
2.59	1.74	1.85	0.97	0.78	0.77	1,85 (7,24)	1,37 (6,09)	n=11426; m=69,6	n=11995; m=66
3.55	3.19	2.87	3	1.59	1.54	4,76 (8,83)	4,61 (8,58)	n=42212; m=47	n=26904; m=37,5
2.73	2.13	2.71	2.15	1.31	1.1	3,68 (8,66)	3,64 (8,62)	n=21006; m=100,9	n=19270; m=100,9
2.02	2.32	1.9	1.9	0.87	1.09	1,96 (6,66)	3,18 (8,31)	n=25633; m=71,9	n=12918; m=64,7
2.86	2.8	2.62	2.4	1.44	1.24	4,20 (8,46)	3,69 (8,29)	n=17150; m=72,3	n=21190; m=85,5

2.15	2.38	3.66	2.91	1.64	1.22	5,21 (9,3)	3,19 (7,54)	n=29397; m=49,7	n=18285; m=48
2.93	2.91	2.79	2.36	1.4	1.09	4,33 (8,79)	2,85 (7,53)	n=28137; m= 64,7	n=16825; m= 66
1.92	1.91	1.63	1.8	0.83	0.78	2,29 (7,75)	1,96 (6,63)	n=29080; m= 55,2	n=24672; m= 65,8
2.44	1.92	2.93	2.94	1.62	1.69	5,25 (9,77)	5,77 (8,85)	n=30110; m= 45,2	n= 48403; m= 43,6
3.5	2.33	3.25	3.01	1.69	1.25	5,96 (10,18)	3,84 (8,46)	n=50563; m=29,5	n=33733; m=38,6
2.52	2.54	1.98	2.88	1.1	1.15	2,82 (7,32)	3,07 (7,58)	n=40448; m=76,4	n=27800; m=70,7
3.39	2.23	1.45	1.44	0.96	0.91	2,49 (7,68)	2,53 (7,86)	n=9342; m=82,5	n=14324; m=95,2
2.21	2.17	3.21	3.76	1.64	1.78	4.78 (9.06)	5.30 (9.43)	n=41860; m=30.9	n=46236; m=40.8
1.23	1.78	2.64	2.87	1.37	1.42	4.20 (8.85)	3.94 (8.50)	n=35082; m=51.7	n=25616; m=80.7
2.13	2.57	2.75	2.98	1.52	1.72	4.36 (8.69)	4.69 (8.63)	n=57991; m=39.5	n=41103; m=56.4
2.24	2.49	2.09	2.03	1.4	1.14	4,35 (9,05)	3,27 (8,37)	n=36882; m=41	n=28602; m=62,2
2.43	3.52	1.54	2.79	0.88	1.53	1.55 (5.8)	4.65 (8.79)	n=8521; m=59	n=17275; m=60
2.58	3.48	2.6	2.38	1.54	1.54	3.85 (7.98)	4.33 (8.47)	n=16869; m=53.3	n=18622; m=39.6
2.1	2.19	2.07	1.88	1.28	1.48	4.42 (9.39)	4.09 (8.86)	n=60357; m=56.4	n=54902; m=60.3
1.49	1.81	3.02	2.84	1.06	1.31	2.42(6.45)	3.49(8.03)	N=15402; M=43.5	n=21296; m=60.8
1.53	2.41	1.53	1.81	1.11	1.24	3.09(8.36)	3.25(8.59)	n=40051; m=42.6	n=43815; m=56.1
1.87	2.18	3.54	2.17	1.65	1.27	4.79(8.85)	3.28(8.08)	n=28220; m=43.9	n=19550; m=70.1
2.74	1.51	1.19	1.46	0.87	0.98	3.57(7.68)	2.73(7.44)	n=14263; m=41.1	n=12.065; m=55.3
2.09	2.11	3.31	2.93	1.27	1.36	2.55(6.77)	2.90(7.09)	n=13588; m=53.2	n=17437; m=53.1
2.7	1.88	2.97	3.2	1.76	1.69	5.64(9.72)	4.84(8.96)	38761; m=56.2	n=40015; m=53.9
2.19	2.33	2.96	2.79	1.63	1.49	5.10(9.14)	4.53(9.06)	n=42800;m=41.3	n=36869; m=51.3

Table S3. Follow-up Ultrasound Data

RBiceps R-T	LBiceps R-T	RQuad-T	LQuad-T	RRF-T	LRF-T	RRF-CA	LRF-CA	RRF-Echoin	LRF-Echoin
1.68	1.61	1.4	1.41	0.7	1	2,24 (7,7)	2,34 (7,54)	n=55743; m=61,1	n=57155; m=41,2
1.54	2.53	2.78	2.81	1.43	1.82	3,72 (7,99)	5,54 (9,29)	n=21745; m=75,6	n=21259; m=82,6
1.36	2.42	1.67	2.41	1.09	1.29	2,73 (8,15)	4,34 (8,94)	n=40474; m=4,5	n=35731; m=44,3
1.01	1.52	1.53	1.86	0.84	0.96	2,83 (8,43)	3,36 (8,9)	n=23369; m=79,7	n=19024; m=50,6
1.19	2.23	1.71	1.96	0.92	1	2,28 (6,53)	2,1 (6,19)	n=30905; m=51,8	n=29205; m=56,4
3.39	2.09	3.11	3.38	1.63	2.03	5,75 (10,03	6,71 (10,06)	n=32021; m=41,5	n=29589; m=54,5
1.4	2.01	2.71	2.76	1.27	1.76	3,77 (8,06)	4,82 (8,83)	n=51848; m=27,8	n=64135; m=33,3
2.51	1.43	2.84	2.68	1.6	1.37	5,87 (9,82)	3,92 (8,79)	n= 25452; m=77,5	n=23002; m= 58,9
2.46	2.21	2.27	1.7	1.19	1.13	3,1 (8,05)	3,31 (8,47)	n=22959; m= 44,7	n=29559; m=43,3
0.93	1.66	1.31	1.22	1.22	1.04	3,06 (7,94)	2,71 (7,94)	n=19871; m=118,7	n=16165; m=110,3
1.23	2.3	1.3	1.96	0.96	1.51	2,55 (7,25)	3,8 (7,63)	n=10304; m=107,6	n=17976; m=88,6
1.55	1.88	1.37	1.03	0.61	0.88	1,33 (5,56)	1,46 (6,02	n=10490; m= 67,5	n= 10789; m=110,6
3.57	3.53	1.99	2.01	0.87	1.06	1,94 (6,21)	2,71 (7,5)	n=11087; m=42,1	n=26372; m=57
2	1.64	1.75	1.86	0.97	1.06	3,2 (8,39)	3,07 (7,99)	n=55006; m=41	n=39974; m=53,3
2.17	1.83	1.73	2.07	1.12	1.06	2,8 (8,42)	2,98 (7,91)	n=23672; m=58,6	n=18509; m=48,5
1.26	1.59	1.07	1.04	0.52	0.82	1,86 (7,96)	2,11 (7,9)	n=22718; m=50,6	n=29109; m=49,7
2.29	1.76	2.08	1.66	1.17	0.8	3,42 (8,35)	1,99 (6,75)	n=44028; m=34,9	n=26822; m=38
1.64	0.58	1.57	0.8	0.77	0.93	1,81 (7,47)	1,57 (8,15)	n=14133; m=71,7	n=26129; m=65,4
2.24	2.96	3.13	2.45	1.68	2	5,04 (8,76)	5,94 (9,31)	n=29845; m=60,8	n=37849; m=83,2
1.91	1.3	1.78	1.47	0.93	1.06	2,86 (8)	2,64 (8,13)	n=25118; m=39,9	n=22600; m=53,6
1.85	1.45	1.68	2.07	0.94	1.27	1,94 (6,99)	3,06 (7,19)	n=18909; m=61	n=25670; m=62,9

2.43	2.72	2.01	1.72	1.24	1.05	3,94 (8,69)	3,78 (8,64)	n= 33591; m= 81,7	n= 35000; m= 74,1
1.83	2.09	2.32	2.42	1.44	1.53	4,2 (8,75)	4,54 (8,86)	n=38579; m=47,5	n=38947; m=37,7
1.69	1.88	1.22	1.21	0.97	0.76	2,39 (8,07)	1,92 (6,77)	n= 19318; m= 71,8	n= 15740; m= 83,1
1.14	1.58	0.98	1.16	0.97	0.62	2,45 (8,04)	1,59 (6,6)	n=22572; m= 44,3	n=21039; m= 41,8
1.96	2.18	2.35	2.32	1.99	1.23	4,46 (8,91)	3,57 (7,98)	n=28502; m=50,1	n= 27644;m= 52,7
1.48	2.08	2.22	1.8	1.32	1.28	3,8 (9,04)	3,66 (8,79)	n=22670; m=37,2	n=30278; m=41,8
1.62	2.73	1.49	1.82	0.85	1.2	2,71 (8,02)	3,6 (8,87)	n=36234; m=70	n=46909; m=76,4
1.69	2.15	1	1.07	0.81	0.78	1,7 (6,79)	2,53 (7,77)	n=9993; m=117,5	n=14594; m=100,2
2.19	2.05	2.11	2.44	1.45	1.62	4.13 (8.92)	4.57 (8.99)	n=35604; m=25.7	n=37725; m=23.8
1.24	1.6	2.73	2.98	1.45	1.41	3.72 (7.98)	4.25 (8.66)	n=21242; m=50	n=25897; m=65.9
2.74	1.66	3.22	2.79	2.08	1.74	4.22 (7.66)	4.38 (8.20)	n=35890; m=29.1	n=60393; m=34.4
2.4	1.74	2.73	2.64	1.52	1.43	4,12 (8,34)	3,82 (8,34)	n=24882; m=40,6	n=23405; m=55,8
2.66	2	1.78	2.53	0.97	1.26	3.20 (8.07)	3.30 (7.77)	n=14687; m=65.2	n=13672; m=64.1
2.78	2.74	2.57	2.42	1.6	1.54	3.76 (8.25)	4.28 (8.73)	n=20357; m=36.3	n=25989; m=37.6
1.48	1.97	2.15	2.43	1.33	1.87	4.85(9.51)	5.19(9.67)	n=64654; m=41.5	n=69379; m=38.4
1.1	1.69	2.2	1.68	1.34	1.48	3.58(8.24)	3.06(9.07)	n=49893; m=49.7	n=41072; m=47.9
1.16	2.22	1	1	1.12	0.66	2.91(7.76)	1.73(7.10)	n=22746; m=41.7	n=22342; m=54.8
1.87	1.59	1.87	2.62	1.07	1.3	3(7.85)	3.49(7.81)	n=26260; m=52.9	n=28443; m=51.8
2.46	1.79	0.7	1.25	0.56	0.68	1(6.43)	1.39(7.59)	n=14431; m=59.3	n=19647; m=53.4
2.05	1.76	2.41	2.14	1.34	1.44	3.55(8.08)	3.66(8.15)	n=29089; m=43.7	n=34345; m=51.6
2.53	2.48	2.35	2.3	1.61	1.49	5.26(9.59)	4.17(8.95)	n=22586; m=45	n=17895; m=43.7
3.01	2.95	2.73	2.96	1.46	1.68	4.04(8.58)	3.95(8.04)	n=33681; m=39.6	n=35059; m=57.8
